# Dr. Bernard Clausen

**Published:** 1914-11

**Authors:** 


Dr. Bernard Clausen, N. Y. Homeopathic, 1888, formerly of Hoboken, N. J., died at his home in Binghamton, September 24, aged 50.

